# *Arabidopsis* LEAFY COTYLEDON1 controls cell fate determination during post-embryonic development

**DOI:** 10.3389/fpls.2015.00955

**Published:** 2015-11-03

**Authors:** Mingkun Huang, Yilong Hu, Xu Liu, Yuge Li, Xingliang Hou

**Affiliations:** ^1^Key Laboratory of South China Agricultural Plant Molecular Analysis and Genetic Improvement, South China Botanical Garden, Chinese Academy of SciencesGuangzhou, China; ^2^University of the Chinese Academy of SciencesBeijing, China

**Keywords:** *Arabidopsis*, LEAFY COTYLEDON1, trichome, cell fate determination, post-embryonic development

## Abstract

*Arabidopsis* LEAFY COTYLEDON1 (LEC1) transcription factor is a master regulator that shapes plant embryo development and post-embryonic seedling establishment. Loss-of-function of *LEC1* alters the cotyledon identity, causing the formation of ectopic trichomes, which does not occur in wild-type seedlings, implying that LEC1 might regulate embryonic cell fate determination during post-embryonic development. To test this hypothesis, we compared the expression of trichome development-related genes between the wild-type and the *lec1* mutant. We observed that transcripts of GLABROUS1 (*GL1*), *GL2*, and *GL3*, genes encoding the positive regulators in trichome development, were significantly upregulated, while the TRICHOMELESS1 (*TCL2*), ENHANCER OF TRY AND CPC1 (*ETC1*), and *ETC2* genes, encoding the negative regulators in trichome development, were downregulated in the *lec1* mutant. Furthermore, overexpression of *LEC1* activated the expressions of *TCL2*, CAPPICE (*CPC*), and *ETC1*, resulting in production of cotyledonary leaves with no or fewer trichomes during vegetative development. In addition, we demonstrated that LEC1 interacts with TCL2 in yeast and *in vitro*. A genetic experiment showed that loss-of-function of *GL2* rescued the ectopic trichome formation in the *lec1* mutant. These findings strongly support that LEC1 regulates trichome development, providing direct evidence for the role of LEC1 in cell fate determination during post-embryonic development.

## Introduction

In higher plants, embryogenesis generally terminates with a dormancy period for future sporophyte growth. Once conditions are favorable, seeds germinate, and undergo post-embryonic development, during which cells acquire specific fates. Extensive studies have shown that the specific cell fate determination during post-embryonic development is precisely controlled by multiple transcription factors (Peris et al., 2010; Perianez-Rodriguez et al., 2014). The plant Nuclear Factor Y (NF-Y) transcription factor LEAFY COTYLEDON1 (LEC1), a master regulator controlling embryogenesis (Meinke, 1992; West et al., 1994; Harada, 2001; Lee et al., 2003), was recently revealed to play a potential role in regulating post-embryonic development (Junker and Baumlein, 2012). *LEC1* is expressed in both developing embryo and post-embryonic seedlings, but not in the vegetative true leaves (Kwong et al., 2003; Warpeha et al., 2007; Le et al., 2010). Loss-of-function of *LEC1* causes a pleiotropic phenotype, including embryo desiccation intolerance and defects in the accumulation of seed storage compounds in developing seeds, as well as cotyledon with trichomes, premature development of vascular tissue and mesophyll cells, and short hypocotyls in early seedlings (Meinke, 1992; Meinke et al., 1994; West et al., 1994; Brocard-Gifford et al., 2003). By contrast, ectopic expression *LEC1* allows seedlings to remain in the embryonic state with an unexpanded cotyledon after germination and is sufficient to induce conversion of true leaves into embryonic structures that lack trichomes (Lotan et al., 1998; Junker et al., 2012). These findings support the multifunctional roles of LEC1 in embryonic and post-embryonic development. Trichomes originate from the epidermal cell layer whose nuclei have undergone multiple rounds of endoreduplication, which then enlarge and expand away from the surface (Hulskamp et al., 1994; Pattanaik et al., 2014). The regulation of trichome formation has been well documented in many studies. The R2R3 MYB transcription factor GLABROUS1 (GL1) was the first identified positive regulator involved in trichome development (Marks and Feldmann, 1989; Oppenheimer et al., 1991). GL1 interacts with the bHLH transcription factor GLABRA3 (GL3), its close homolog ENHANCER OF GLABRA3 (EGL3), and the WD40-repeat factor TRANSPARENT TESTA GLABRA1 (TTG1), resulting in the formation of the MYB-bHLH-WD-repeat trichome-positive transcription complex (Zhao et al., 2008). This protein complex subsequently induces trichome formation by activating the expression of the downstream gene *GLABRA2 (GL2)*, encoding a homeodomain-leucine zipper (HD-Zip) transcription factor (Rerie et al., 1994; Morohashi et al., 2007). Contrastingly, a small single-repeat R3 MYB protein family, including TRICHOMELESS1 (TCL1), TCL2, TRIPTYCHON (TRY), CAPPICE (CPC), ENHANCER OF TRY AND CPC1 (ETC1), and ETC2, plays redundant roles in the negative regulation of trichome development. These proteins compete with GL1 for binding to GL3/EGL3, resulting in the formation of an inactive protein complex that fails to activate *GL2* expression and thus represses trichome formation (Wang and Chen, 2014; Zhou et al., 2014).

Trichome formation is considered a specific characteristic that distinguishes true leaves from cotyledons in *Arabidopsis* (Chandler, 2008). A hypothesis for the ectopic trichome formation on the cotyledons of the *lec1* mutant is that the loss-of-function of *LEC1* might result in the disturbance of embryonic cell fate determination, allowing the precocious post-germination events in which cotyledons are partially converted into true leaves (West et al., 1994; Chandler, 2008). However, the mechanism by which LEC1 functions in cell fate determination during post-embryonic development remains elusive.

Given that LEC1 mutation causes remarkable ectopic trichome growth on cotyledons, the trichome-related genes might serve as good candidates to study the function of LEC1 in the post-embryonic development phase. Here, we demonstrated that LEC1 represses trichome cell differentiation during post-embryonic development by regulating trichome-related genes. In addition, LEC1 was shown to interact with TCL2, a repressor of trichome development, which indicated that LEC1 might couple with other transcription factors to co-regulate trichome formation. A genetic experiment showed that loss-of-function of *GL2* rescued the ectopic trichome development phenotype of the *lec1* mutant. These results strongly supported the view that *LEC1* functions in cell fate determination during the post-embryonic phase, providing new insights into *LEC1*’s role beyond the embryogenesis.

## Materials and Methods

### Plant Materials and Growth Conditions

All *Arabidopsis* plants used in this study are in the Col genetic background. The mutant *gl2* was described previously (Wang et al., 2010). The mutant *lec1-4* (Salk_095699) was obtained from the *Arabidopsis* Biological Resource Center (http://www.arabidopsis.org). The estradiol-inducible *pER10:LEC1-MYC* transgenic line was obtained by kanamycin selection after transformation (Mu et al., 2008). The double mutant was generated by genetic crossing. After surface sterilization, seeds were sown on the half-strength Murashige and Skoog medium containing 0.8% agar and incubated at 4°C in the dark for 3 days. The seedlings were then transferred to the growth chamber at 22°C under long day conditions (16 h light/8 h dark). For estradiol treatment, 10 μM estradiol was added to the half-strength Murashige and Skoog medium plate and dimethyl sulfoxide, the solvent for estradiol, served as the mock treatment. After treatment, these seedlings were harvested for further analyses.

### Microscopy

The formation of trichomes on the cotyledons and leaves was observed and photographed using a LEICA M165C stereoscope (Leica, Wetzlar, Germany).

### Statistical Analysis

Five-day-old seedlings were observed to calculate frequency of the appearance of ectopic trichomes on cotyledons. At least 15 Col and 50 *lec1-4* seedlings were used in each experiment. Data represent mean ± SD of three independent repeats. To calculate the frequency of different trichome types on *lec1-4* cotyledons, at least 50 trichomes were analyzed in each experiment. Data represent the mean ± SD of seven independent repeats.

### Gene Expression Analysis

Total RNA was extracted from 5-day-old *Arabidopsis* seedlings using the Plant RNA Kit (OMEGA, Atlanta, GA, USA), following the manufacturer’s instructions. One microgram of total RNA was used for the reverse transcription reaction. The first-strand cDNA was synthesized using the M-MLV reverse transcriptase (Promega, Madison, WI, USA), according to the manufacturer’s instructions. Quantitative real-time PCR was performed using the LightCycler480 system (Roche, Basel, Switzerland) in a total volume of 10 μl, with 0.25 μl of each primer (10 μM), 1 μl of cDNA product and 5 μl of SYBR Premix ExTaq (Takara, Tokyo, Japan). The PCR program included an initial denaturation step at 94°C for 1 min, followed by 40 cycles of 10 s at 94°C and 1 min at 60°C. Each sample was quantified at least in triplicate and normalized using the *TUB2* gene as the internal control. Primers used in this study are listed in Supplementary Table S1.

### Yeast Two-Hybrid Assay

The full-length coding sequences of *LEC1* and *TCL2* were amplified and cloned into vectors pGBKT7 and pGADT7 (Clontech, Palo Alto, CA, USA), respectively. Primers are listed in Supplementary Table S1. Yeast two-hybrid assays were performed following the manufacturer’s instructions of the Yeastmaker Yeast Transformation System 2 (Clontech). In brief, a single colony of yeast *AH109* was incubated at 30°C overnight (OD_600_ > 1.0). The cells were harvested by centrifugation and then resuspended in 25 mL ddH_2_O. After re-harvesting the cells by centrifugation, a 1.5-ml sterile 1× Tris-EDTA/LiAc solution was added to prepare yeast-competent cells. For the yeast two-hybrid assay, the bait (0.5 μg) and/or prey (0.5 μg) plasmids with 0.1 mg of carrier DNA were co-transformed into the yeast-competent cells using Polyethylene glycol/LiAc solution. After incubation at 30°C for 30 min, 70 μL dimethyl sulfoxide was added and incubation continued at 42°C for 15 min. The cells were centrifuged and washed using Tris-EDTA solution. After transformation, the yeast cells were grown on SD/-Trp/-Leu/-His/-Ade medium for the interaction test.

### Pull-Down Assay

To produce glutathione-S-transferase (GST)-TCL1, GST-TCL2, GST-CPC, GST-ETC1, His-LEC1, and His-GFP recombinant proteins, the full-length coding sequences of the tested genes were cloned into vector pGEX-4T-1 (Pharmacia, Piscataway, NJ, USA) or pQE30 (QIAGEN, Dusseldorf, Germany), respectively. Primers are listed in Supplementary Table S1. These constructs were transformed into the *Escherichia coli* Rosetta strain DE3 (Novagen, Billerica, MA, USA). One hundred milliliters of (OD_600_ ≈ 0.5) Rosetta strains harboring various vectors were incubated at 16°C for 16 h with 0.1 mM isopropyl β-D-1-thiogalactopyranoside. The soluble GST fusion proteins were purified using glutathione sepharose beads (Amersham Biosciences, Piscataway, NJ, USA), while His fusion proteins were purified using Ni- nitrilotriacetic acid agarose beads (QIAGEN), according to the manufacturer’s instructions, respectively. For pull-down assays, 2 μg of His-LEC1 or His-GFP were incubated with immobilized GST or GST fusion proteins in the binding buffer (50 mM Tris-HCl, pH 8.0, 100 mM NaCl, and 1 mM EDTA) at 4°C for 4 h. After washing with binding buffer three times, proteins retained on the beads were subsequently resolved by sodium dodecyl sulfate polyacrylamide electrophoresis and detected with an anti-His antibody (GBI, Beijing, China).

## Results

### Ectopic Trichome Formation on the Cotyledons of the *lec1* Mutant

**FIGURE 1:**
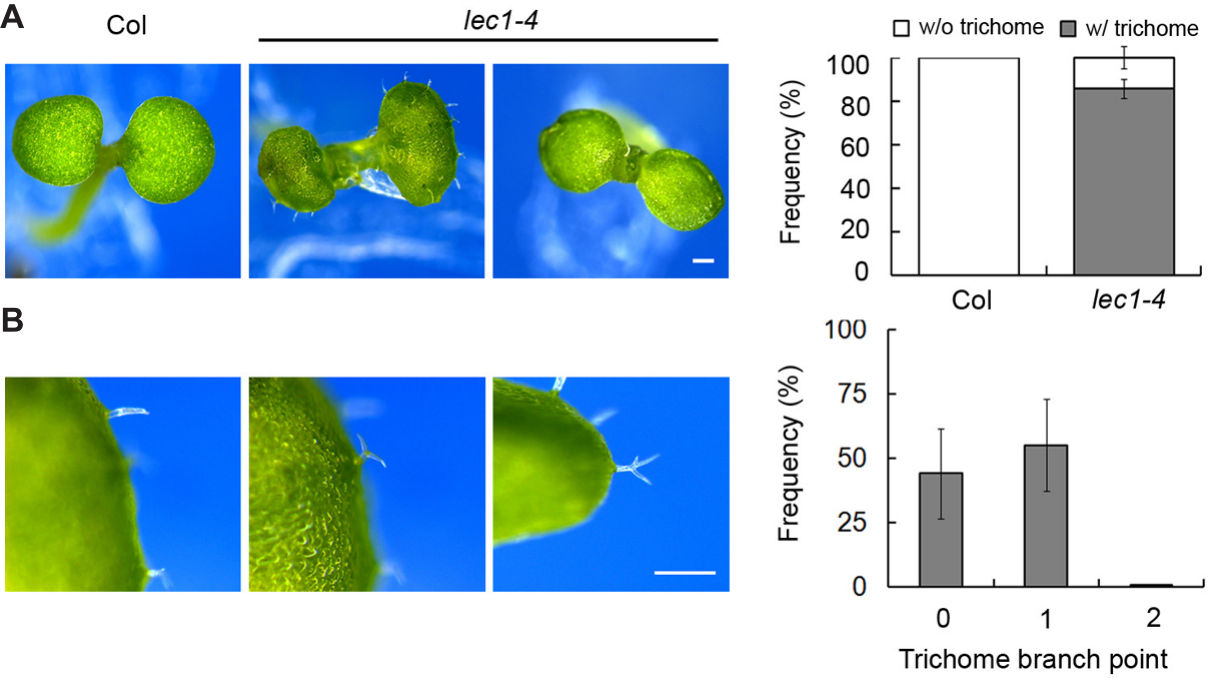
**Ectopic trichome formation on the *lec1-4* mutant cotyledons. (A)** Comparison of 5-day-old Col wild-type and *lec1-4* mutant cotyledons. Bar = 0.5 mm. The right panel indicates the frequency of seedlings with (w/) or without (w/o) ectopic trichomes on their cotyledons. **(B)** Light micrographs of three types of trichome branch on *lec1-4* mutant cotyledons. Bar = 0.5 mm. The right panel indicates the frequency of different types of trichomes. 0 indicates no branch on a trichome; 1 indicates that the trichome has two branches; 2 indicates a trichome with three branches.

Extensive studies have shown that LEC1 serves as a key regulator in embryo development, which is consistent with its main expression in the developing seed (Kwong et al., 2003). However, recent observations suggested a potential role of LEC1 in post-embryonic cell differentiation, including hypocotyl elongation and the formation of vascular tissue, mesophyll cells and trichomes (Warpeha et al., 2007; Junker and Baumlein, 2012; Junker et al., 2012; Supplementary Figure S1A). Serving as a general model system in the study of cell fate determination in *Arabidopsis* (Yang and Ye, 2013), we focused on trichome formation to reveal the function of LEC1 in post-embryonic development. A desiccation-tolerant leaky allele *lec1-4* whose mature seeds germinate normally (unpublished data) was used in this study. Consistent with previous reports, the wild-type cotyledons were glabrous, while ∼85% of *lec1* cotyledons produced a varied number of ectopic trichomes (**Figure 1A**; Supplementary Table S2). Moreover, unlike the mostly three-branched trichomes developed on true leaves, the trichomes on *lec1* cotyledons were mainly unbranched (∼44%) or two-branched (∼55%), and rarely three-branched (less than 1%; **Figure 1B**; Supplementary Table S3). These observations implied incomplete conversion of the *lec1* cotyledons into true leaves. In addition, there was no significant difference observed in trichome numbers or the morphology of rosette leaves between the wild-type and *lec1*, which is consistent with the observation that no *LEC1* transcripts accumulated in the vegetative tissues (Supplementary Figures S1A,B).

### Misregulation of Trichome-Related Genes in the *lec1* Mutant

**FIGURE 2:**
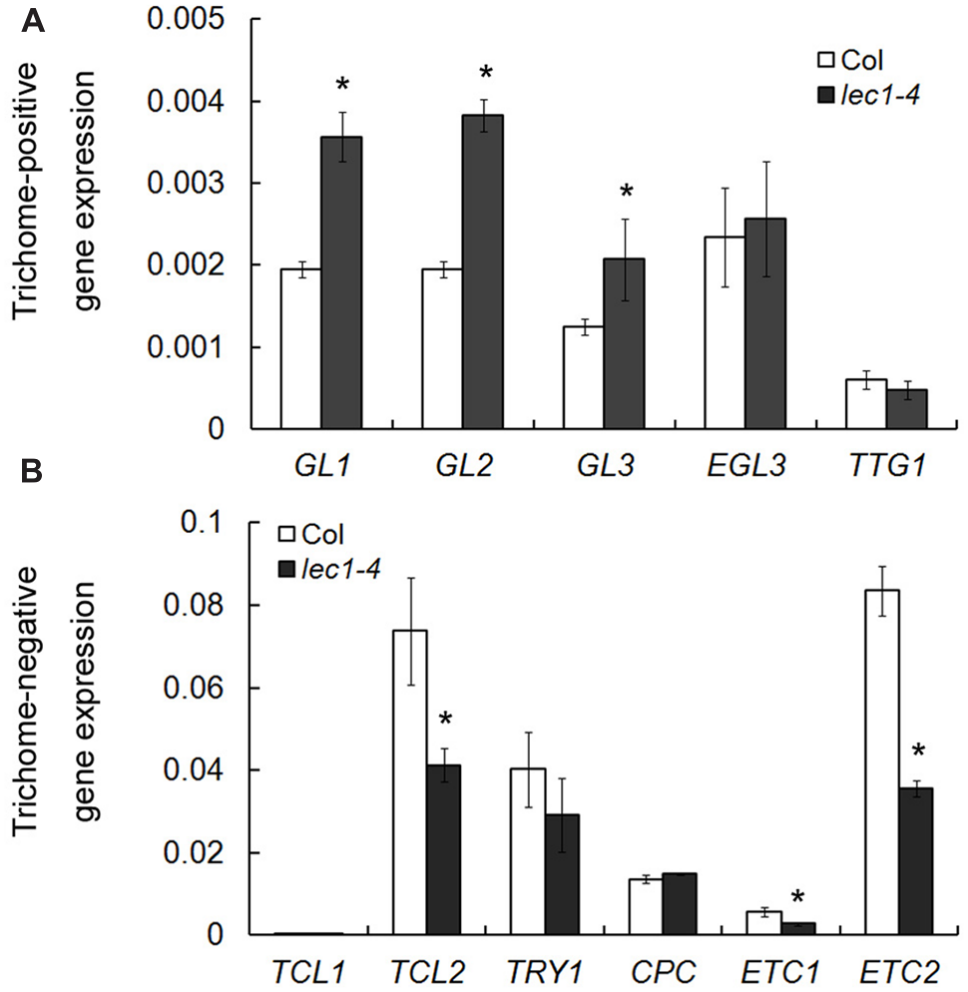
**Misregulation of trichome-related genes in the *lec1-4* mutant seedlings. (A)** The expression analysis of trichome-positive genes in 5-day-old Col wild-type and *lec1-4* mutant seedlings. **(B)** The expression analysis of trichome-negative genes in 5-day-old Col wild-type and *lec1-4* mutant seedlings. *TUB2* was used as an internal control. Asterisks indicate significant differences between Col and *lec1-4* mutant (*p* < 0.05, by Student’s *t*-test).

Trichome formation on leaves has been well studied during the past two decades and a number of trichome-related genes were identified, which could be divided into two groups: trichome-positive and -negative genes (Yang and Ye, 2013). To examine whether LEC1’s involvement in the inhibition of trichome formation on cotyledons occurs by mediating the regulation of trichome-related genes, five trichome-positive genes, and six trichome-negative genes were selected for quantitative real-time PCR analysis in wild-type and *lec1* seedlings. Interestingly, among the trichome-positive genes, the expressions of *GL1*, *GL2*, and *GL3* were significantly upregulated in the *lec1* mutant compared with the wild-type, while those of *EGL3* and *TTG1* showed no change (**Figure 2A**). Moreover, the expressions of the trichome-negative genes *TCL2*, *ETC1*, and *ETC2* were significantly downregulated in the *lec1* mutant compared with the wild-type, while those of *TRY* and *CPC* showed little change. The *TCL1* transcript was hardly detected in either the wild-type or *lec1* seedlings (**Figure 2B**). These results indicated that the ectopic trichome formation might be caused by misregulation of the trichome-related genes in the *lec1* mutant.

### Overexpression of *LEC1* Activates Several Trichome-Negative Transcription Factors

**FIGURE 3:**
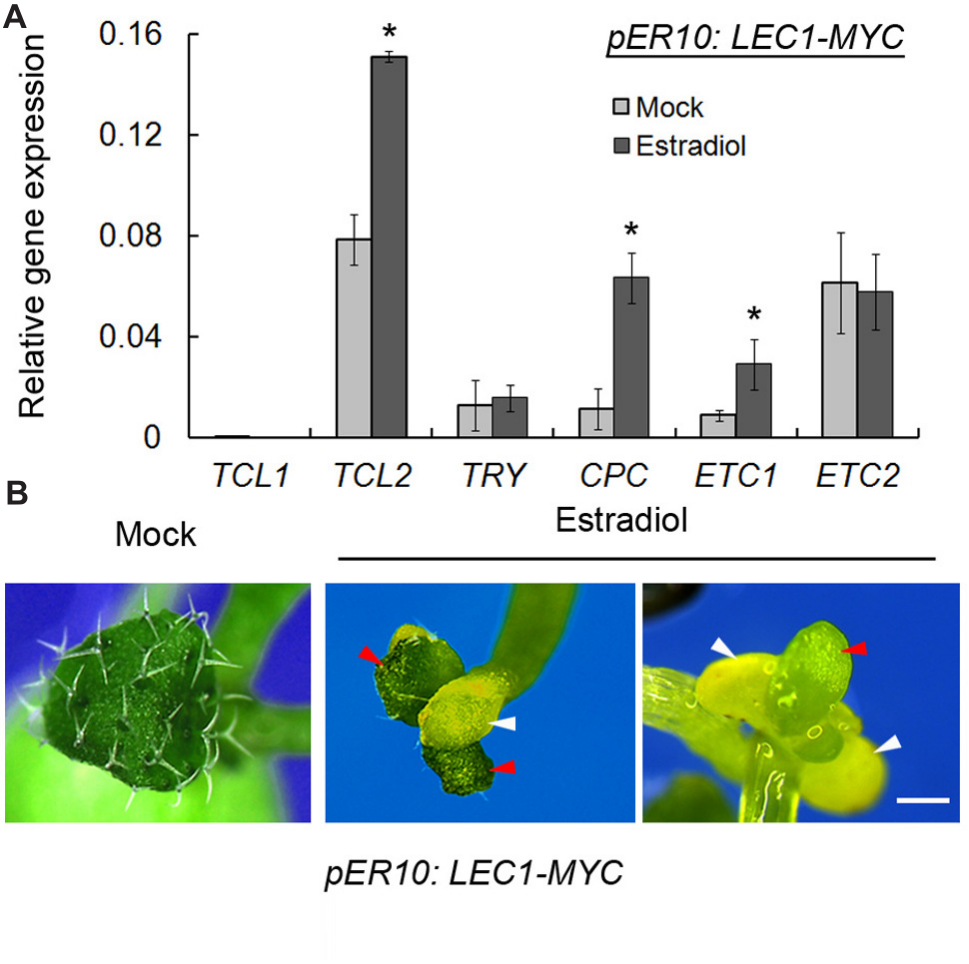
**Induced *LEC1* overexpression suppresses trichome formation. (A)** The expression analysis of trichome-negative genes in 5-day-old *pER10:LEC1-MYC* transgenic seedlings with 10 μM estradiol or mock treatment. *TUB2* was used as an internal control. Asterisks indicate significant differences between estradiol and mock (*p* < 0.05, by Student’s *t*-test). **(B)** Induced *LEC1* overexpression promotes conversion of true leaves into embryonic structures. Fourteen-day-old *pER10:LEC1-MYC* transgenic seedlings with 10 μM estradiol or mock treatment were used for phenotype observation. Red arrowheads indicate rosette leaves; white arrowheads indicate cotyledons. Bar = 1 mm.

Given that loss-of-function of *LEC1* resulted in misregulation of trichome-related genes, we wondered whether *LEC1* overexpression could activate or repress these genes. *LEC1* overexpression, driven by the 35S promoter, caused complex phenotypes and embryonic lethality (Lotan et al., 1998). Thus, instead, we employed an inducible transgenic plant harboring *pER10:LEC1-MYC*, in which *LEC1* expression could be significantly induced by estradiol (Mu et al., 2008; Supplementary Figure S2). Unexpectedly, the trichome-positive genes *GL1*, *GL2*, *GL3*, *EGL3*, and *TTG1* were not regulated by induced LEC1 (data not shown). Nevertheless, overexpression of *LEC1* activated the expressions of *TCL2*, *CPC*, and *ETC1*, but had no effect on those of *TCL1*, *TRY*, and *ETC2* (**Figure 3A**), which revealed the role of LEC1 in the regulation of trichome-negative genes. A previous study reported that *TCL2*, *CPC*, and *ETC1* overexpression lines all presented a glabrous leaves phenotype (Wang and Chen, 2014); therefore, we asked whether ectopic *LEC1* expression affects trichome formation on true leaves. The investigation demonstrated that cotyledons of *pER10:LEC1-MYC* under either mock or estradiol treatment displayed a similar glabrous phenotype to the wild-type (data not shown). Strikingly, under long-term induction by estradiol (14 days), the *pER10:LEC1-MYC* seedlings developed fewer trichomes and cotyledon-like rosette leaves (**Figure 3B**). Together with a previous report that the single-repeat R3 MYB quintuple mutant *try cpc etc1 etc3 tcl1* produced ectopic trichomes on cotyledons similar to the *lec1* mutant (Wang et al., 2008), our observations supported the hypothesis that LEC1 suppresses trichome formation via activation of *TCL2*, *CPC*, and *ETC1* expression.

### LEC1 Physically Interacts with TCL2 *In Vitro* and in Yeast

**FIGURE 4:**
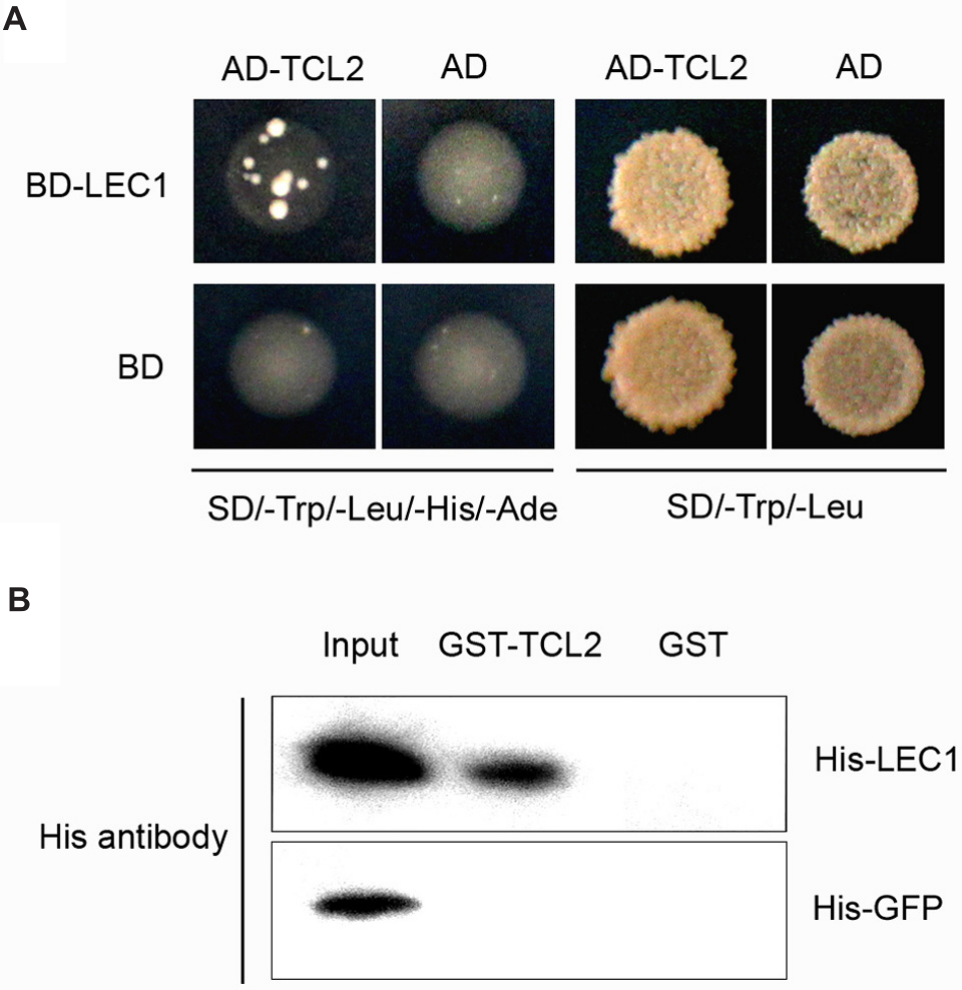
**TCL2 physically interacts with LEC1 in yeast and *in vitro*. (A)** Yeast two-hybrid assays showing that TCL2 interacts with LEC1. Transformed yeast cells were grown both on SD/-Trp/-Leu/-His/-Ade and SD/-Trp/-Leu medium. **(B)** A pull-down assay showing the direct interaction between His-LEC1 and GST-TCL2 fusion proteins *in vitro*. His-LEC1 or His-GFP protein was incubated with immobilized GST or GST-TCL2 proteins and the immunoprecipitated fractions were detected by an anti-His antibody.

LEAFY COTYLEDON1 belongs to the NF-Y family, whose members interact with other transcription factors to co-regulate target gene expression (Yamamoto et al., 2009). To investigate whether LEC1 couples with cofactors to mediate trichome formation, we performed a yeast two-hybrid screening assay. Interestingly, the trichome transcriptional repressor TCL2 was observed to interact with LEC1 in yeast (**Figure 4A**). We then performed a pull-down assay to confirm the interaction between LEC1 and TCL2 using purified GST- and His-tagged proteins (Supplementary Figure S3). As expected, GST-TCL2 interacted with His-LEC1, but not with the His-GFP protein, while GST alone did not precipitate either of the His-tagged proteins (**Figure 4B**). TCL2 and its homologs TCL1, TRY, CPC, ETC1, and ETC2 exert a master role in repressing trichome formation during the whole life of plants; therefore, we further tested the binding between LEC1 and these homologous proteins. The pull-down results showed that LEC1 also interacted with TCL1, CPC, and ETC1 *in vitro* (Supplementary Figure S4); however, their bindings were not observed in the yeast two-hybrid assay, probably because of weak interactions in yeast (data not shown). These results indicated that, besides regulating the expression of the single-repeat R3 MYB genes, LEC1 might mediate trichome formation via direct protein interactions with these repressors, a result that requires further investigation.

### GL2 Genetically Acts Downstream of LEC1

**FIGURE 5:**
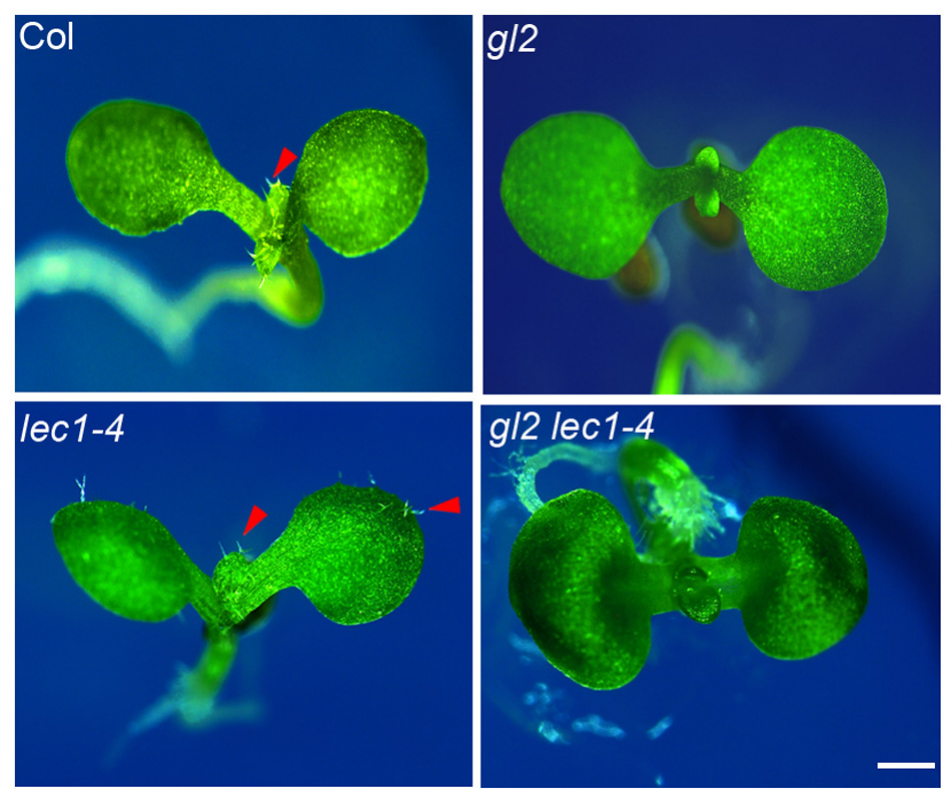
**Loss-of-function of *GL2* rescues the phenotype of ectopic trichome formation on *lec1* cotyledons.** Ten-day-old Col, *gl2*, *lec1*, and *gl2 lec1* seedlings were used for the phenotype observation. A red arrowhead indicates the trichomes. Bar = 1 mm.

*GLABRA2* functions as the key transcription activator in trichome development, whose expression is activated by the trichome-positive MYB-bHLH-WD and repressed by the trichome-negative single-repeat R3 MYB-bHLH-WD transcription complexes (Zhao et al., 2008). Loss-of-function of *GL2* completely abolished trichome formation on the first pair of true leaves in early seedlings (**Figure 5**). The elevated *GL2* expression in the *lec1* (**Figure 2A**) implied an epistatic effect of *GL2* on *LEC1* in trichome formation on cotyledons. To test this hypothesis, we created a *gl2 lec1* double mutant and found that similar to *gl2*, the *gl2 lec1* double mutant displayed glabrous true leaves (**Figure 5**). In contrast, the *gl2* mutation repressed the ectopic trichome formation on cotyledons of *lec1* seedlings remarkably (**Figure 5**), confirming the primary role of GL2 in trichome formation, regardless of the developmental stage. These results strongly supported the hypothesis that *GL2* is required for *LEC1*-mediated repression of trichome formation on cotyledons.

## Discussion

Post-embryonic development is a vital transition state from embryonic status to vegetative growth, during which seedlings sense various environmental cues, such as light and temperature, to enable young sporophyte survival. At this stage, cell fate determination occurs to promote post-embryonic organogenesis that arises from the primary meristems, during which the visible features, such as cotyledons extension, hypocotyl elongation, and the emergence of true leaves with trichomes, can be observed in seedlings. Studies have shown that multiple endogenous factors, including phytohormones and transcription regulators, are involved in post-embryonic seedling development (Perianez-Rodriguez et al., 2014). LEC1, the master regulator in embryogenesis, was recently reported to be involved in light-mediated post-embryonic hypocotyl elongation (Junker and Baumlein, 2012). In this study, we provide molecular evidence that *LEC1* and its genetic downstream partner *GL2* are required for the repression of trichome formation on embryo-derived cotyledons, the characteristic that distinguishes them from true leaves. Mutation of *LEC1* resulted in the misregulation of several trichome-related genes and thus triggered trichome formation on cotyledons. By contrast, ectopic LEC1 activates the expression of the single-repeat *R3 MYB* genes and induces embryonic leaves with few or no trichomes. Furthermore, the results indicated that LEC1 might regulate trichome formation by interacting with other transcription factors (e.g., TCL2 and its homologs). Taken together, our findings support the hypothesis that LEC1 plays an essential role in cell fate determination during the post-embryonic development.

The fact that *LEC1* is expressed in both developing seeds and post-embryonic seedlings, combined with the pleiotropic phenotype of the *lec1* mutant, supports the multifunctional roles of *LEC1* in plant development (Junker and Baumlein, 2012; Supplementary Figure S1A); however, less is known about the downstream proteins of the LEC1-mediated regulatory process. A recent report indicated that LEC1 might regulate directly a number of genes involved in light, phytohormone signaling, and embryogenesis, including several bHLH and MYB transcription factors (Junker et al., 2012). However, using a chromatin immunoprecipitation assay, we observed that LEC1 does not target the selected trichome-related genes directly (data not shown), indicating that these genes might not act immediately downstream of LEC1. Instead, we observed that LEC1 interacts with the trichome development-related transcription repressors TCL1, TCL2, CPC, and ETC1 *in vitro*. *TCL1* was reported to be highly expressed in developing seeds and its protein targets the *GL1* promoter, while *TCL2* is highly expressed in cotyledons, rather than in developing seeds, implying distinct roles of these two homologous genes in trichome regulation (Wang et al., 2007; Gan et al., 2011). The interactions between LEC1 and these repressors provide a possible mechanism by which LEC1 regulates embryonic cell fate determination spatio-temporally, via coupling with different transcription factors. Nevertheless, whether LEC1 and TCL2 together determine the post-embryonic trichome initiation requires further investigation.

Phytohormones serve as crucial endogenous integrators to mediate plant development by controlling precisely exact cell division and differentiation (Lumba et al., 2010; Vanstraelen and Benkova, 2012). Gibberellin (GA) has been reported to act as a positive endogenous factor to promote trichome formation, because the GA biosynthesis defective mutant *ga1* displays fewer trichomes on its leaves (Perazza et al., 1998; Qi et al., 2014). LEC2 (Stone et al., 2001) and FUSCA3 (FUS3, Keith et al., 1994), two B3 domain-containing transcription factors, also defined as the *LEC* genes, promote *GL1* expression via direct activation of the GA biosynthesis gene, *ATGA3ox2*. When crossed to *ga1*, the ectopic trichome formation on *lec2* and *fus3* cotyledons was completely suppressed (Curaba et al., 2004). However, the ectopic trichomes were not abolished in *lec1 ga1* double mutant, which suggested that LEC1-mediated trichome formation is independent of GA (Curaba et al., 2004). Given that *LEC2* and *FUS3* act as potential downstream candidates of LEC1 (Junker et al., 2010), GA’s effect on the function of LEC factors might be under the control of a complicated regulatory network. Abscisic acid is also involved in LEC1-mediated trichome formation. A previous study reported that the conversion of leaves into cotyledon-like structures is enhanced in the *LEC1* overexpression line when coupled with abscisic acid treatment (Junker et al., 2012). In addition, a number of abscisic acid-responsive genes were shown to be the direct targets of LEC1 (Junker et al., 2012). Nevertheless, the role of phytohormones in LEC1-mediated cell fate determination remains elusive and requires further investigation.

Accurate control of *LEC1* expression is critical for plants to regulate cell fate determination during development. Epigenetic regulation plays key roles in the exclusion of *LEC1* in the vegetative growth stage. PICKLE, a CHD3 chromatin-remodeling factor, represses *LEC1* transcriptional activation in vegetative tissue by modifying histone methylation. In the *pickle* mutant, the seedling root produced an embryonic structure, termed the “PICKLE root”, caused by the ectopic expression of *LEC1* (Ogas et al., 1997, 1999; Li et al., 2005). In addition, the polycomb group proteins CLF and SWN were demonstrated to couple with PICKLE to repress the conversion from vegetative tissue to an embryonic structure (Chanvivattana et al., 2004; Aichinger et al., 2009). Histone deacetylases (e.g., HDA6 and HDA19) also contribute to the repression of embryonic properties by regulating several embryogenesis regulators, including *LEC1* (Tanaka et al., 2008). In addition to these epigenetic factors, some transcription factors, such as VP1/ABI3-LIKE and MYB115/118, were also reported as negative regulators of *LEC1* (Suzuki et al., 2007; Wang et al., 2009; Jia et al., 2013). Embryonic development originates from the zygote after fertilization in higher plants (Lau et al., 2012). High expression during embryogenesis indicates that LEC1 is released from the suppressing environment of the vegetative tissue, thus allowing it to controls embryonic and post-embryonic cell fate determination.

## Conclusion

In this study, we revealed that LEC1 interacts with other transcription factors and regulates trichome-related genes expression to repress the trichome formation on cotyledons. These findings provide evidence that supports the hypothesis that LEC1, probably by integrating phytohormone signals and epigenetic regulation, mediates cell fate determination during the post-embryonic development phase, providing new insights into the role of LEC1 beyond embryogenesis.

## Accession Numbers

Sequence data from this article can be found in the *Arabidopsis* Genome Initiative database under the following accession numbers: *LEC1* (AT1G21970), *GL1* (AT3G27920), *GL2* (AT1G79840), *GL3* (AT5G41315), *EGL3* (AT1G63650), *TCL1* (AT2G30432), *TCL2* (AT2G30424), *TRY* (AT5G53200), *CPC* (AT2G46410), *ETC1* (AT1G01380), *ETC2* (AT2G30420), and *TUB2* (AT5G62690).

## Author Contributions

MH and XH designed the research. MH, YH, XL, and YL performed experiments. MH, YH, and XH analyzed data. MH and XH wrote the paper.

## Conflict of Interest Statement

The authors declare that the research was conducted in the absence of any commercial or financial relationships that could be construed as a potential conflict of interest.
