# Supplementary material for: Arabidopsis LEAFY COTYLEDON1 controls cell fate determination during post-embryonic development
Source: Front Plant Sci. 2015 Nov 3;6:955. doi: 10.3389/fpls.2015.00955 (PMC4630568; doi:10.3389/fpls.2015.00955)
Supplement: Supplementary file 1 [file Data_Sheet_1.PDF]

Supplementary Material

Arabidopsis LEAFY COTYLEDON1 controls cell fate determination during post-embryonic development

Mingkun Huang,[1,2] Yilong Hu,[1,2] Xu Liu,[1] Yuge Li,[1] and Xingliang Hou[1*]

**** Correspondence:*** *Corresponding Author: houxl@scib.ac.cn*

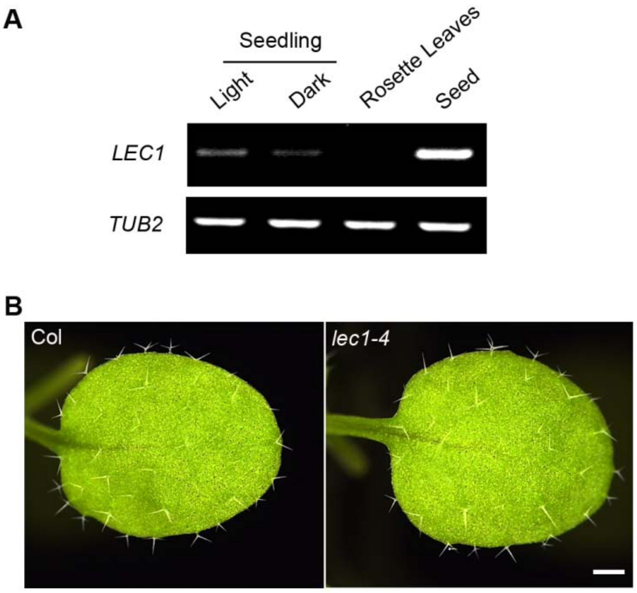

Supplemental Figure S1. LEC1 is not involved in trichome formation on rosette leaves. (A) *LEC1* mRNA accumulated in developing seeds and 5-day-old light- and dark-grown seedlings but not in rosette leaves. *TUB2* was used as an internal control. **(B)** Trichome phenotype of the first pair of rosette leaves in Col wild type and *lec1-4* mutant showed no obvious distinction. Bar = 1 mm.

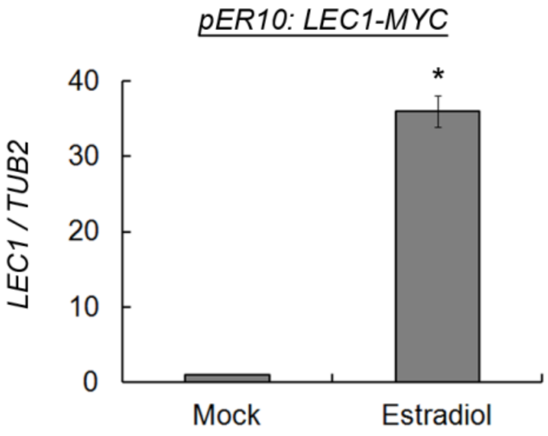

Supplemental Figure S2. The chemical inducer estradiol significantly induces ectopic *LEC1* expression. 5-day-old *pER10:LEC1-MYC* transgenic seedlings grown in the light with 10 μM estradiol or mock treatment were used for gene expression analysis. *TUB2* was used as an internal control. Asterisk indicates significant difference between estradiol and mock ($p < 0.05$, by Student's *t*-test).

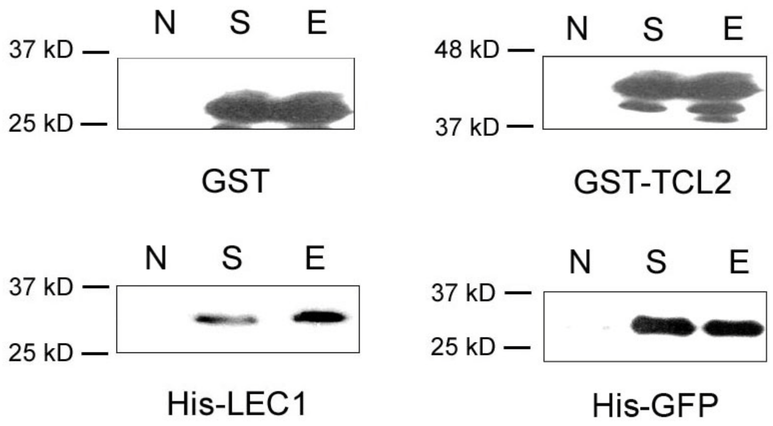

Supplemental Figure S3. Western blot detection of GST- and His-tagged proteins. N, no IPTG; S, soluble GST- or His-tagged fusion proteins supernatant; E, elution component.

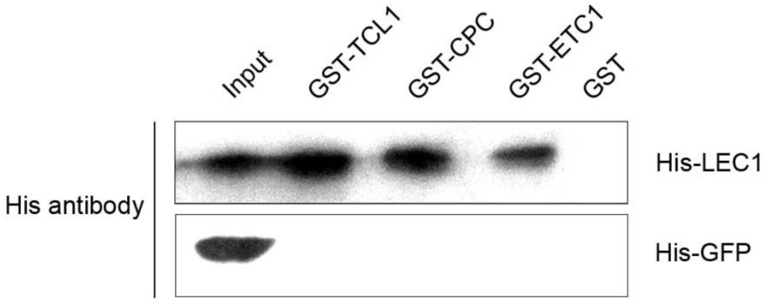

Supplemental Figure S4. Pull-down assay showing direct interaction between His-LEC1 and GST-TCL1, GST-CPC or GST-ETC1 fusion proteins *in vitro*. His-LEC1 or His-GFP protein was incubated with immobilized GST or GST-fusion proteins, and immunoprecipitated fractions were detected by anti-His antibody.

Supplemental Table S1. List of primers used in this Study.

Primers for quantitative real-time PCR

Gene name	Primers (5’-3’)
LEC1	F:AAACCTATGGAGGAAATGG
	R:TAATGGAAGACGAAGAGCC
GL1	F: TGGAACCGCATCGTCAGA
	R: ATTGCCGAGGAGCTTGTG
GL2	F: CAGGCGAACCTATGTGGC
	R: CATGAAACTCTGGGCAAGT
GL3	F: AATGAGGTGGTTATTGAGC
	R: ATTCCTGGTGTCGCTATT
EGL3	F: TGAGGAGGAAAGAGCATC
	R: TATCCGTTAGACCAGCAT
TTG1	F: ATTACAACAACCGCATCG
	R: AAGGAGGACTGAACATTAGC
TRY	F: GGTGATAGGTGGGATTTG
	R: TGAGGCTTGGTATGTTTG
CPC	F: TGTTCCGAAGAGGTGAGT
	R: CGTGTTTCATAAGCCAATA
ETC1	F: AACCATTGTTGCCTCTTC
	R: TAAATCCCACCTTTCACC
ETC2	F: TTGTCGGTAATAGGTGGG
	R: ACGTCGTCGTTTGTGAGA
TCL1	F: CACTGTCATAAACAACCCAA
	R: CACGTCCCACCACTCTTC
TCL2	F: AACCGTCTTCGTCACCTT
	R: CTCGTCCTACCACTCTTCC
TUB2	F: ATCCGTGAAGAGTACCCAGAT
	R: AAGAACCATGCACTCATCAGC

Primers for constructs in yeast two-hybrid assays

Gene name	Primers (5’-3’)
BD-LEC1	F:AATTCATATGATGGAACGTGGAGCTCCCT
	R: CGCGGATCCTCACTTATACTGACCATA
AD-TCL2	F: CCGGAATTCATGGATAACACCAACCGT
	R: CCGCTCGAGTTAAGGAGGAGAAATAGA

Primers for constructs in pull-down assays

Gene name	Primers (5’-3’)
GST-TCL1	F: CCGGAATTCATGGATAACACAAACCGT
	R: CCGCTCGAGTCATTTGTGGGAGAAATA
GST-TCL2	F: CCGGAATTCATGGATAACACCAACCGT
	R: CCGCTCGAGTTAAGGAGGAGAAATAGA

GST-CPC	F: CCGGAATTCATGTTTCGTTCAGACAAG
	R: CCGCTCGAGTCATTTCCTAAAAAAGTC
GST-ETC1	F: CCGGAATTCATGAATACGCAGCGTAAG
	R: CCGCTCGAGTCAACGTAATTGAGATCT
His-LEC1	F:CGCGGATCCATGGAACGTGGAGCTCCCTT
	R:ACGCGTCGACCTTATACTGACCATAATGGT
His-GFP	F: CGCGGATCCATGAGTAAAGGAGAAGAAC
	R: ACGCGTCGACTTTGTATAGTTCTACCAT

Supplemental Table S2 Frequency of seedlings with (w/) or without (w/o) ectopic trichomes on cotyledons.

		Seedlings w/o trichomes	Seedlings w/ trichomes	Frequency of seedlings w/o trichomes(%)	Frequency of seedlings w/ trichomes(%)
Col	Experiment1	15	0	100.00	0.00
	Experiment2	15	0	100.00	0.00
	Experiment3	15	0	100.00	0.00
	Mean	--	--	100.00	0.00
	SD	--	--	±0.00	±0.00
lec1-4	Experiment1	8	46	14.81	85.19
	Experiment2	10	42	19.23	80.77
	Experiment3	5	51	8.93	91.07
	Mean	--	--	14.32	85.68
	SD	--	--	±5.16	±5.17

Supplemental Table S3 Frequency of different types of trichomes on cotyledons.

lec1-4	Trichome branch point			Total number of trichomes	Frequency (%) Trichome branch point		
	0	1	2		0	1	2
Experiment1	48	32	0	80	60.00	40.00	0.00
Experiment 2	24	36	1	61	39.34	59.02	1.64
Experiment 3	13	58	1	72	18.06	80.56	1.39
Experiment 4	30	40	0	70	42.86	57.14	0.00
Experiment 5	29	55	1	85	34.12	64.71	1.18
Experiment 6	34	42	1	77	44.16	54.55	1.30
Experiment 7	40	16	0	56	71.43	28.57	0.00
Mean	--	--	--	--	44.28	54.93	0.79
SD	--	--	--	--	±17.33	±16.79	±0.75
